# Polyphosphate Degradation in Stationary Phase Triggers Biofilm Formation via LuxS Quorum Sensing System in *Escherichia coli*

**DOI:** 10.1371/journal.pone.0050368

**Published:** 2012-11-30

**Authors:** Mariana Grillo-Puertas, Josefina M. Villegas, María R. Rintoul, Viviana A. Rapisarda

**Affiliations:** Instituto Superior de Investigaciones Biológicas (Consejo Nacional de Investigaciones Científicas y Técnicas-Universidad Nacional de Tucumán), and Instituto de Química Biológica “Dr Bernabé Bloj” (Universidad Nacional de Tucumán), San Miguel de Tucumán, Tucumán, Argentina; Tulane University, United States of America

## Abstract

In most natural environments, association with a surface in a structure known as biofilm is the prevailing microbial life-style of bacteria. Polyphosphate (polyP), an ubiquitous linear polymer of hundreds of orthophosphate residues, has a crucial role in stress responses, stationary-phase survival, and it was associated to bacterial biofilm formation and production of virulence factors. In previous work, we have shown that *Escherichia coli* cells grown in media containing a critical phosphate concentration >37 mM maintained an unusual high polyP level in stationary phase. The aim of the present work was to analyze if fluctuations in polyP levels in stationary phase affect biofilm formation capacity in *E. coli*. Polymer levels were modulated by the media phosphate concentration or using mutant strains in polyP metabolism. Cells grown in media containing phosphate concentrations higher than 25 mM were defective in biofilm formation. Besides, there was a disassembly of 24 h preformed biofilm by the addition of high phosphate concentration to the medium. These phenotypes were related to the maintenance or re-synthesis of polyP in stationary phase in static conditions. No biofilm formation was observed in *ppk^−^ppx^−^* or *ppk^−^ppx^−^*/*ppk^+^* strains, deficient in polyP synthesis and hydrolysis, respectively. *luxS* and *lsrK* mutants, impaired in autoinducer-2 quorum sensing signal metabolism, were unable to form biofilm unless conditioned media from stationary phase wild type cells grown in low phosphate were used. We conclude that polyP degradation is required for biofilm formation in sufficient phosphate media, activating or triggering the production of autoinducer-2. According to our results, phosphate concentration of the culture media should be carefully considered in bacterial adhesion and virulence studies.

## Introduction

In clinical and industrial settings, bacteria are found predominantly forming biofilms and not as planktonic cells, such as those typically studied in the laboratory [1], [2]. Biofilms are defined as a complex cell assemblages enclosed in an adherent matrix which exhibit channels and pillars that are thought to allow the exchange of nutrients and wastes [1], [3], [4]. Biofilm formation is regulated by environmental conditions, such as nutrient availability [1], [5] and ionic strength [6], [7]. A model for biofilm development proposes that this phenomenon is initiated by the attachment of individual cells to a surface, followed by their migration and replication to form microcolonies to eventually produce the mature biofilm [8], [9]. These structures, which are generally hundreds of microns in depth, are difficult to eradicate by conventional techniques, such physical or chemical treatments, and cause problems in many natural, environmental, clinical, and industrial settings [3], [10], [1], [11], [12], [13].

Bacteria regulate gene expression in response to changes in cell population density by a process called quorum sensing (QS) [14]. This bacterial mechanism involves the release and detection of chemical signal molecules called autoinducers. Detection of minimal threshold stimulatory concentration of these molecules enables bacteria to distinguish between low and high cell population density and to control target gene expression in response to fluctuations in cell number [14]–[19]. Among the extracellular signal molecules, autoinducer-2 (AI-2) mediates interspecies communication and facilitates regulation of bacterial behaviors such as biofilm formation and virulence [20]. In *Escherichia coli*, the above mentioned AI-2, is derived from the precursor (S)-4,5-dihydroxy-2,3-pentanedione (DPD), which is synthesized by LuxS, encoded by the *luxS* gene [21]. Also, *E. coli* pathogenesis is regulated in a QS-dependent manner in response to indole production [22]. Indole is synthesized by TnaA, a tryptophanase encoded by *tnaA* gene [23]. Mtr (encoded by *mtr* gene), a high-affinity tryptophan permease, is the main conduit for indole import in *E. coli* [24].

Inorganic polyphosphate (polyP) is a linear chain of tens or many hundreds of phosphate residues linked by high-energy phosphoanhydride bonds, which is found in every cell in nature: bacterial, archaeal, fungal, protozoan, plant, and animal [25], [26]. Some of the polyP functions are the substitution for ATP in kinase reactions; reservoir of phosphate; chelation of divalent metals; capsule production in bacteria; and regulatory roles in growth, development, stress, and nutrients deprivation [25], [26]. In bacteria, polyP is usually accumulated during exponential phase of growth, and degraded at the beginning of stationary phase. The main enzymes associated with polyP metabolism are the polyphosphate kinases (PPK, encoded by *ppk* gene), responsible for the polyP synthesis from ATP; and the exopolyphosphatase (PPX, encoded by *ppx* gene), responsible for the polyP hydrolysis [27]–[29]. The different roles of polyP have been inferred from mutant cells lacking polyphosphate kinase (PPK), deficient in polyP synthesis. For instance, using *ppk* mutants of several microorganisms such as *Pseudomonas aeruginosa, E. coli*, *Salmonella enterica* serovar Dublin, *Vibrio cholerae*, *Bacillus cereus* and *Porphyromonas gingivalis*, polyP formation was shown to be critical for attributes such as motility, quorum sensing, biofilm formation, resistance to oxidative, osmotic, heat, nutritional and alkaline stress, and stationary-phase survival [25], [27], [30]–[37]. Although the importance of polyP in various bacterial phenotypes has been reported, the precise molecular mechanisms by which polyP enacts specific functions, as well as the primary and secondary effects of poly-P accumulation, are still not understood in even the best-characterized bacterial species.

We have previously shown that *E. coli* cells grown in shacked media containing a critical phosphate concentration >37 mM maintained an unusual high polyP level in stationary phase (up to 72 h) [38]. Here, we found that high phosphate media impaired biofilm formation, and this phenotype was related with the maintenance of polyP in stationary phase in static growth. The present study is a first step towards the investigation of how polyP levels fluctuations induce quorum sensing signals involved in biofilm formation.

## Materials and Methods

### Bacterial strains, growth conditions and media

Bacterial strains used in this study are listed in **Table 1**. For inocula preparation, isolated colonies were grown aerobically at 37°C with linear shaking in MT or MT+P medium. MT minimal medium contains per liter of distilled water: 0.272 g KH_2_PO_4_ (corresponding to 2 mM), 5.8 g NaCl, 3.7 g KCl, 0.15 g CaCl_2_.2H_2_O, 1.1 g NH_4_Cl, 0.142 g Na_2_SO_4_, 12.1 g Tris (Tris [hydroxymethyl] aminomethane), 0.27 mg FeCl_3_, and 0.2 g MgSO_4_.7H_2_O. MT media with 40 mM phosphate buffer pH 7 was designated as MT+P [42]. MT cultures supplemented at 24 or 48 h with 40 mM phosphate buffer pH 7 were named as MT+24P and MT+48P, respectively. Also, 24 h MT+P cells shifted to MT fresh medium were represented as MT+P→24MT. When indicated, minimal medium was prepared with phosphate concentrations other than 2 or 40 mM. Phosphate buffer was prepared with sodium phosphate salts (Sigma). In all experiments, 0.4% glucose and 0.1% tryptone were used as carbon and nitrogen sources, respectively. When required, antibiotics were used: 40 µg mL^−1^ of ampicillin or 50 µg mL^−1^ of kanamycin. Viability was determined by counting the CFU on LB-agar plates incubated at 37°C for 24 h.

**Table 1:** *E. coli* strains and plasmid used in this work.

Strains or plasmid	Relevant genotype or description	Source or reference
MC4100	araD, Δlac, rpsL, flbB, deoC, ptsF, rbsR, relA1	[39]
BW25113	K-12 derivative, Δ(araD-araB)567, ΔlacZ4787(::rrnB-3), λ^−^, rph-1, Δ(rhaD-rhaB)568, hsdR514	CGSC
MG1655	F^−^, λ^−^, rph-1	CGSC
C	*E. coli (Migula) Castellani and Chalmers*	ATCC
LSB022	MC4100 (*ppkppx*::Km)	[38]
LSB022/pBC29	LSB022/pBC29((*ppkppx*::Km/*ppk^+^*, Ap)	This study
JW2662-1	BW25113 (*luxS::Km*)	CGSC
JW3686-7	BW25113 (*tnaA::Km*)	CGSC
JW3130-1	BW25113 (*mtr::Km*)	CGSC
MG1655 *lsrK^−^*	MG1655 (*lsrK^−^*)	[40]
pBC29	(*ppk^+^*, Ap)	[41]

### Quantification of biofilm formation

Biofilm formation was assayed on the basis of the ability of cells to adhere and grow on 96-well polystyrene microtiter plates [8]. Overnight stationary phase cultures in MT or MT+P were diluted to an OD_560_ = 0.1 (corresponding to CFU mL^−1^ from 5 to 6×10^7^) with fresh medium and grown in statics conditions at 30°C in microtiter plates for 24, 48 or 72 h. After removing the unattached cells and rinsed the plates three times with deionized water, quantification of attached cells was performed as follows. Two hundred microliters of a 0.1% crystal violet solution was added to each well, and the plates were incubated at room temperature for 30 min in dark. Then, the wells were rinsing again three times with water. Crystal violet stained attached cells was extracted with 200 µl of 95% ethanol. Absorbances of the adherent and non-adherent cells were measured at 595 nm and 560 nm, respectively (SpectraMax Plus384 Absorbance Microplate Reader, US). Six replicates were performed for each condition studied in each experiment.

### Conditioned media experiments

Conditioned media (CM) are spent media obtained from bacterial cultures by two sequential centrifugation at 15 000 r.p.m. and a 0.2 µm filtration. 24 h MT growing cells were shifted to CM and incubated for further 24 h before biofilm quantification.

### Measurements of polyP level

Intracellular polyP was measured in cell suspensions growing in static conditions, using a DAPI (4′,6-diamidino-2-phenylindole) based fluorescence approach [43]. Cells were washed and resuspended in buffer T (100 mM Tris HCl, pH 7.5). 17 µM DAPI (Sigma) was added to cuvettes containing cell suspensions in buffer T, with SDS and chloroform for cell permeabilization, at an OD_560_ = 0.02. After 5 min of agitation at 37°C, the DAPI fluorescence spectra (excitation, 415 nm; emission, 445 to 650 nm) were recorded using an ISS PCI spectrofluorometer (Champaign, IL). Fluorescence (in arbitrary units) of the DAPI-polyP complex at 550 nm was used as a measure of intracellular polyP since fluorescence emissions from free DAPI and from DAPI-DNA are minimal at this wavelength [43].

### Statistical analysis

Data were subjected to analysis of variance (ANOVA) followed by Tukey's test with Statitix 9.0 Analytical Software 2008 for Windows (USA). Differences at *p*-value of 0.05 were considered significant.

## Results

### High phosphate concentration impairs biofilm formation

**Figure 1:**
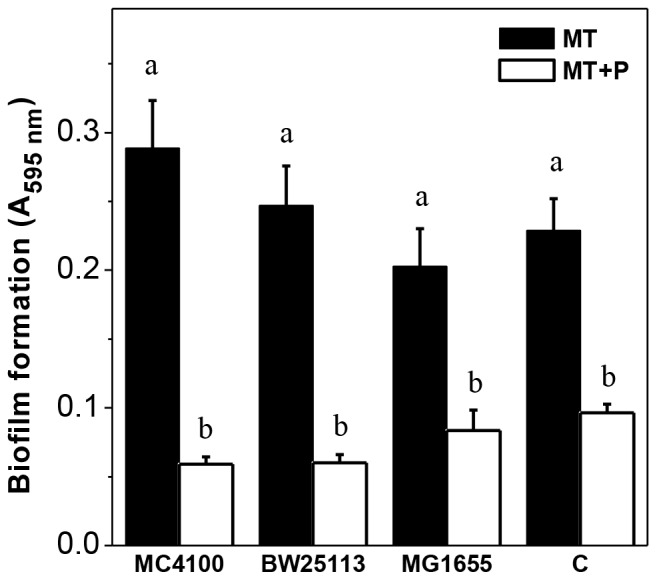
Biofilm formation in MT and MT+P media. *E. coli* wild type strains were grown in static conditions during 48 h at 30°C in the indicated media. The biofilm amount was determined by the crystal violet assay. Results represent the mean ± SD of four independent experiments. Different letters indicate significant differences according to Tukey's test with a *p*-value of 0.05.

**Figure 2:**
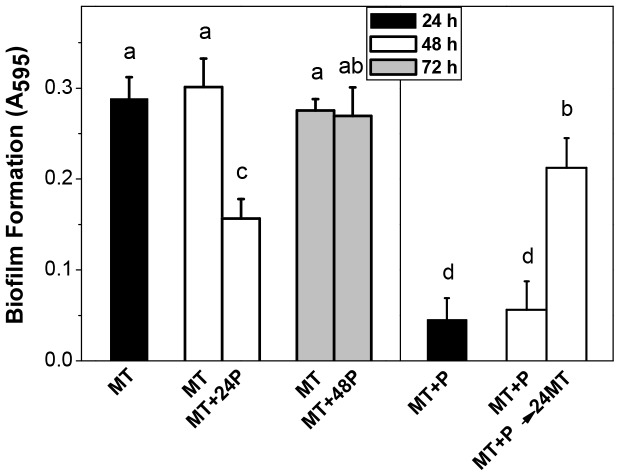
Biofilm formation after changes of phosphate concentration in stationary phase. Biofilm formation by MC4100 was measured at indicated times of growth in MT and in MT with the addition of 40 mM phosphate buffer pH 7 at 24 h (MT+24P) or at 48 h (MT+48P) (**left panel**), or in MT+P and in MT+P culture switched to fresh MT at 24 h (MT+P→24MT) (**right panel**). Result represents the mean ± SD of at least three independent experiments performed in triplicate. Different letters indicate significant differences according to Tukey's test with a *p*-value of 0.05.

The ability of different *E. coli* strains to form biofilm was studied in sufficient (MT, 2 mM) or high (MT+P, 40 mM) phosphate media. In all wild type strains tested, the biofilm formed at 48 h by MT+P cells was around 2.5 fold lower than that of cells grown in MT (**Fig. 1**). Furthermore, when 40 mM phosphate buffer was added to a 24 h MT culture (MT+24P), disassemble of the preformed biofilm was achieved. This effect was not observed when the Pi addition was carried out at 48 h (MT+48P) (**Fig. 2** **, left panel**). On the other hand, when non-biofilm forming MT+P cells were switched to fresh MT medium at 24 h (MT+P→24MT), the adherence at 48 h increased significantly (**Fig. 2** **, right panel**). Since similar results were achieved with the other wild type strains (data not shown), following studies were performed using MC4100.

### Biofilm formation dependency with polyP levels fluctuations

**Figure 3:**
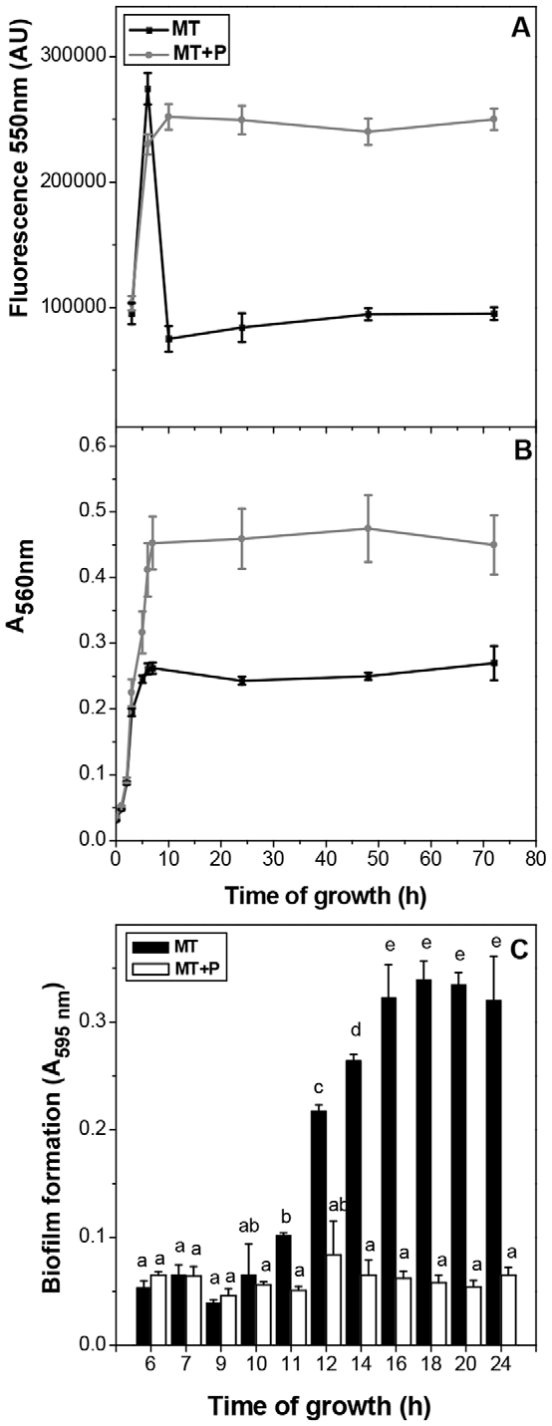
PolyP levels and growth in static cultures. The fluorescence intensity in arbitrary units (AU) of DAPI-polyP complex (excitation λ = 415 nm, emission λ = 550 nm) (**A**) and A_560 nm_ (**B**) were measured during the growth curve of MC4100 in static conditions in the indicated media. Biofilm formation was followed at the indicated times and media (**C**). Data represent the mean ± SD of at least four independent experiments. Different letters indicate significant differences according to Tukey's test with a *p*-value of 0.05.

In order to analyze if the impairment of biofilm formation in high phosphate medium was related to intracellular polyP, the polymer levels were measured in cells grown in static conditions at 30°C. **Fig. 3** shows the values obtained at different times of growth in MT and MT+P media. In exponential phase, polyP levels increased independently of the growth media. In stationary phase, a high polymer level (AU = 256000±1960) was maintained in MT+P, whereas in MT medium an abrupt decrease was observed, reaching to minimum detectable values at 10 h (**Fig. 3A**). Under our growth conditions, the OD_560 nm_ at 48 h in MT+P was higher than in MT (**Fig. 3B**). It should be noted that biofilm formation in MT started at 12 h of growth, after the drop of polyP levels, reaching the maximum value from 16 h (**Fig. 3A and C**).

In cells from experimental approach shown in **Fig. 2**, polyP levels were measured 24 h after the media modification. In MT+24P, a recovery of polyP level was observed (AU = 245000±1840), while in MT+48P polyP remained low (AU = 94562±3215) as in MT stationary cells. In MT+P→24MT, there was a drop in polyP levels (AU = 96562±4515).

**Figure 4:**
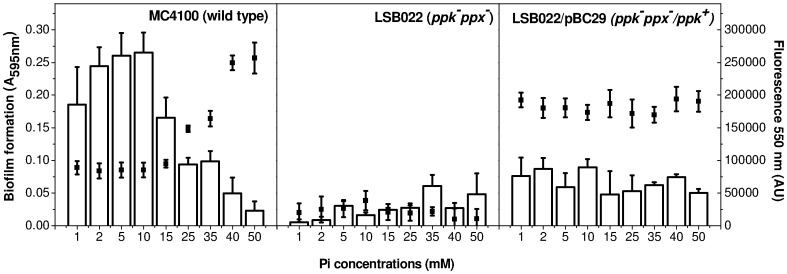
PolyP levels and biofilm formation in different Pi concentrations media. The biofilm amount (white bars) and DAPI-polyP fluorescence (black dots) were determined at 48 h in the indicated *E. coli* strains grown in static conditions in MT medium modified with the indicated Pi concentrations. Data represent the mean ± SD of at least three independent experiments.

PolyP levels and biofilm formation capacity were tested at 48 h in MC4100, LSB022 (*ppk^−^ppx^−^*) and LSB022/pBC29 (*ppk^−^ppx^−^*/*ppk^+^*) cells grown in media supplemented with a wide range of phosphate concentrations. **Fig. 4** shows that in MC4100, both biofilm formation and intracellular polyP levels were dependent on phosphate concentration. In a range between 1 and 10 mM phosphate, both low polyP levels and high biofilm formation were observed. On the contrary, when phosphate was above 25 mM, high intracellular polyP levels were detected and biofilm formation was impaired. In an additional experiment, mixing 24 h cultures from MT and MT+P media (**Fig. S1**), biofilm formation was modified by the media proportion used, similarly to data of MC4100 in **Fig. 4**. In any of the phosphate concentrations tested, biofilm formation was observed in LSB022 and LSB022/pBC29 strains, being the polyP level undetectable in *ppk^−^ppx^−^* deficient strain and high in the complemented strain (**Fig. 4**). For each mutant strain, similar polymer levels to that of **Fig. 4** were obtained during the whole growth curve (not shown).

### AI-2 trigger biofilm formation in MT cells

**Figure 5:**
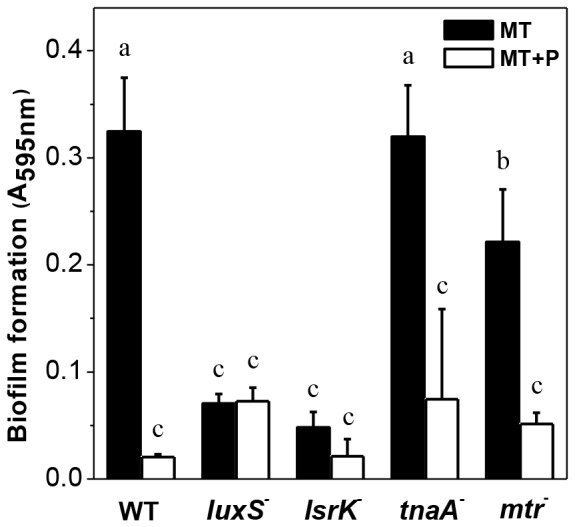
Biofilm formation by quorum sensing mutants. The biofilm amount was determined at 48 h in the indicated strains grown in static conditions in MT or MT+P media. Different letters indicate significant differences according to Tukey's test with a *p*-value of 0.05.

Biofilm formation capacity was analyzed in QS deficient strains, lacking *luxS* (gene encoding for AI-2 synthesis), *lsrK* (gene encoding for AI-2 phosphorylation), *tnaA* (gene encoding for indol synthase) or *mtr* (gene encoding for indol receptor). As shown in **Fig. 5**, indol mutants show similar biofilm phenotypes than that of the wild type in MT and MT+P. However, *luxS* and *lsrK* mutants were unable to form biofilm in MT medium, contrarily to the wild type. Despite this difference, it is noted that the polyP profiles for *luxS^−^* and wild type cells were similar (**Table 2** and **Fig. 3**, respectively).

**Table 2:** PolyP levels in *luxS^−^* strain in static conditions.

	PolyP level (AU)*	
	MT	MT+P
**6 h**	258500±10540 a	245861±10120 a
**24 h**	75000±11500 b	235468±9245 a
**48 h**	84652±6856 b	254896±11486 a

* Fluorescence 550 nm. Different letters indicate significant differences according to Tukey's test with a *p*-value of 0.05.

### Extracellular AI-2 is associated to polyP degradation

**Figure 6:**
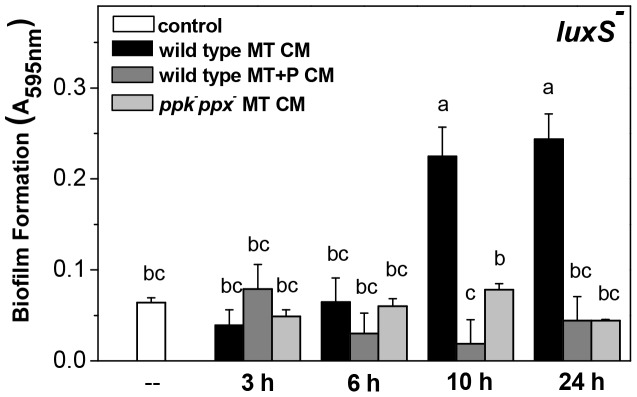
Biofilm formation by *luxS* mutant in conditioned media. *luxS^−^* cells growing in MT medium during 24 h were reinoculated in wild type MT CM or MT+P CM and in *ppk^−^ppx^−^* MT CM from different growth times, as indicated. Biofilm formation was determined 24 h after the shift to CM. 48 h MT cultures were used as control (–). Different letters indicate significant differences according to Tukey's test with a *p*-value of 0.05.

Biofilm formation was quantified in *luxS* mutant shifted to conditioned media (CM). CM were obtained from MT and MT+P wild type and MT *ppk^−^ppx^−^* cultures at different times of growth (3, 6, 10 and 24 h). The deficiency in biofilm formation by *luxS^−^* strain was recovered only when 10 and 24 h wild type MT CM were used (**Fig. 6**). Besides, only 24 h wild type MT CM but not *luxS^−^* MT CM, induced biofilm formation by *ppk^−^ppx^−^* or *ppk^−^ppx^−^*/*ppk^+^* cells (**Table 3**), indicating that extracellular AI-2 is present only in MT CM from stationary phase wild type cultures.

**Table 3:** Biofilm formation after reinoculation in CM.

	Biofilm formation (A_595 nm_)*		
Strains	Control	wild type MT CM	*luxS^−^* MT CM
**LSB022 (** ***ppk^−^ppx^−^*** **)**	0.06223±0.024 a	0.154±0.0124 b	0.0525±0.0235 a
**LSB022/pBC29 (** ***ppk^−^ppx^−^/ppk^+^*** **)**	0.0845±0.0254 a	0.3015±0.0712 c	0.0456±0.0123 a

* Biofilm formation was determined 24 h after the cells transference to CM. 48 h MT cultures were used as control. Different letters indicate significant differences according to Tukey's test with a *p*-value of 0.05.

## Discussion

In many bacterial species, biofilm formation seems to be promoted by non-optimal growth conditions or even by cellular stress [44]. In our experimental conditions, biofilm formation by *E. coli* occurred in MT medium, where stationary phase cells were particularly vulnerable to stress conditions as described by Schurig-Briccio *et al*. [45]. In sufficient phosphate media, biofilm formation from 12 h of growth was associated to the decrease of polyP levels at early stationary phase, while in high phosphate media, the impairment in biofilm formation was correlated to the maintenance of high polyP levels. Variations of intracellular polyP levels, induced by changes of Pi concentration, were feasible in cultures up to 24 h. These phosphate dependent modulations of polyP levels were responsible for differences in biofilm amount.

PolyP has been described to be critical for mechanisms such as motility, quorum sensing and biofilm formation [33]–[35]. Kim *et al.* [46] proposed that the presence of polyP in *P. aeruginosa* exponential phase is important for regulatory purposes, for instance, participating in the production of exopolysaccharides such as alginate. Our data obtained with LSB022 (*ppk^−^ppx^−^*) support that polyP synthesis is necessary for cell adherence. In addition, impairment in biofilm formation was observed in LSB022/pBC29 (*ppk^−^ppx^−^*/*ppk^+^*) strain. In the Gram positive bacterium *Bacillus cereus*, Shi *et al*. [47] observed that both *ppk* and *ppx* mutants were defective in motility and biofilm formation. They considered that maintenance of intracellular concentration of polyP within a narrow range, as that of wild type, might be essential for biofilm formation effectiveness [47]. However, we propose that polyP degradation carried out at the beginning of stationary phase, rather than its presence or maintenance, is relevant to trigger adherence process in *E. coli*. This affirmation is based on the different biofilm phenotypes observed in wild type cells, promoted by fluctuations of intracellular polyP levels modulated by media phosphate concentration.

We consider that autoinducer-2 is involved in biofilm formation in MT, since *luxS* mutant was unable to form biofilm in this medium. However, biofilm formation in *luxS* mutant was induced by CM obtained from MT stationary phase wild type cultures (polyP degrading cells), but not using CM obtained from non-degrading polyP cells. These data suggest that polyP degradation and/or the released Pi or energy may be involved in the regulation of AI-2 synthesis. Eisenbach [48] has described that polyP might be a substitute for ATP in the phosphorilation of chemotaxis systems proteins or that phospho-PPK might directly transfer phosphate (phosphorylation by crosstalk). Further studies are necessary to elucidate the signal produced after polyP degradation responsible for AI-2 synthesis.

Taken together, we conclude that polyP degradation is required for biofilm formation in sufficient phosphate media, activating or triggering the production of autoinducer-2. PPX could be provided as an additional target to discover antibiotics against pathogenic *E. coli* cells. Our findings on the relationship between exogenous phosphate concentration, biofilm formation and polyP level may help to elucidate the importance of phosphate and/or its metabolites as signals for virulence responses in pathogens.

## Supporting Information

Figure S1 **Biofilm formation in mixed cultures.** Biofilm formation by MC4100 was measured 24 h after mixing 24 h MT and MT+P cultures in the indicated proportions. Result represents the mean +/− SD of at least two independent experiments performed in duplicate. Different letters indicate significant differences according to Tukey's test with a *p*-value of 0.05.
(TIF)
