# Supplementary figures and images for: Polyphosphate Degradation in Stationary Phase Triggers Biofilm Formation via LuxS Quorum Sensing System in Escherichia coli
Source: PLoS One. 2012 Nov 30;7(11):e50368. doi: 10.1371/journal.pone.0050368 (PMC3511525; doi:10.1371/journal.pone.0050368)

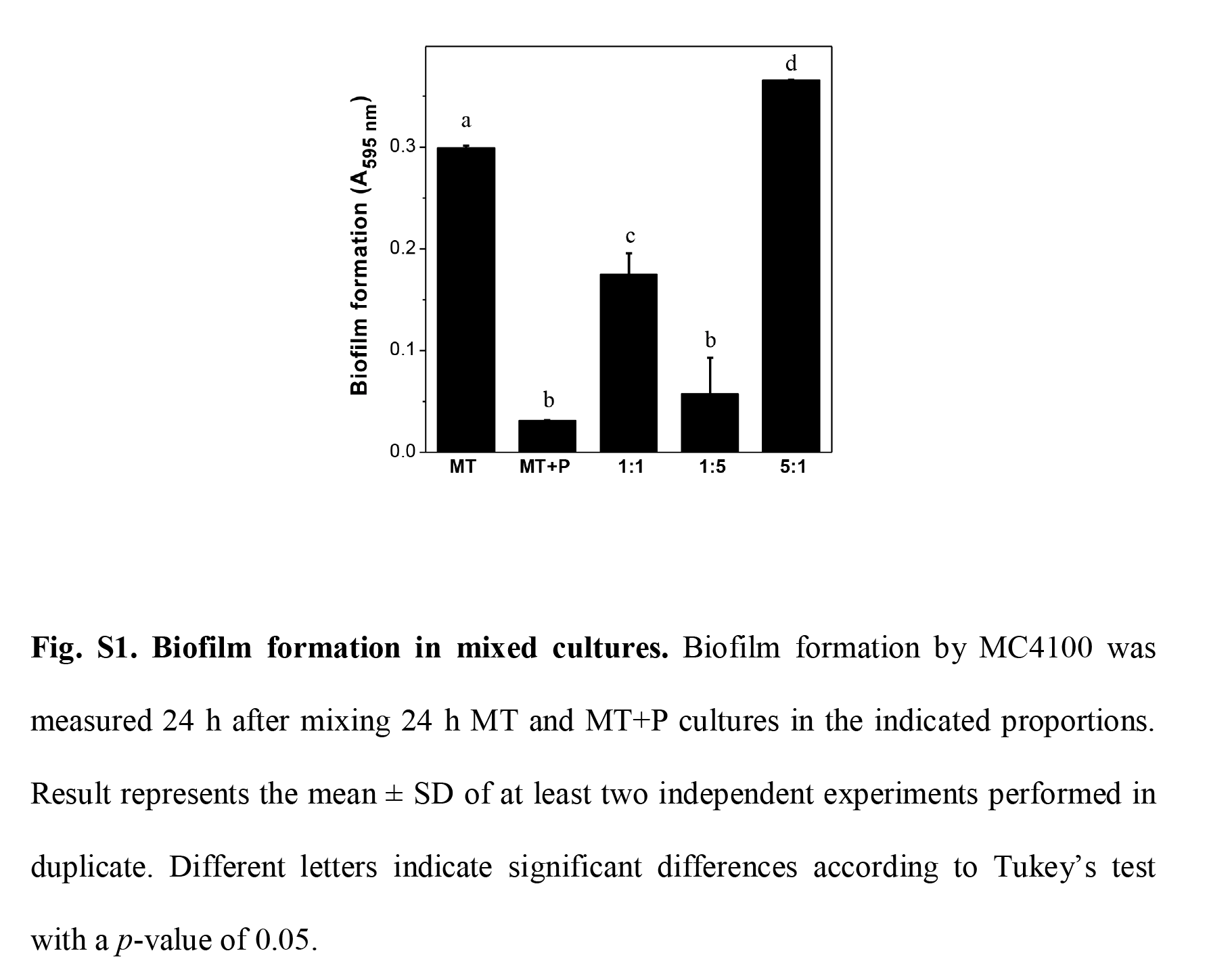

Supplement: Figure S1 — Biofilm formation in mixed cultures. Biofilm formation by MC4100 was measured 24 h after mixing 24 h MT and MT+P cultures in the indicated proportions. Result represents the mean +/− SD of at least two independent experiments performed in duplicate. Different letters indicate significant differences according to Tukey's test with a p-value of 0.05. (TIF) [file pone.0050368.s001.tif]
